# Breathing and Bicycling

**Published:** 1896-12

**Authors:** John O. Roe

**Affiliations:** Rochester, N. Y. 28 North Clinton Street


### BREATHING AND BICYCLING.

### THE ILL EFFECTS OF MOUTH-BREATHING UPON THE THROAT AND LUNGS, AND THE IMPORTANCE OF FREE NASAL RESPIRATION IN THE EXERCISE OF WHEELING.[1]

By JOHN O. ROE, M. D., Rochester, N. Y.

THE function of respiration is one of the most important, if not the most important, of the vital functions over which man in any degree has control. It is, therefore, the one that is most easily and most frequently perverted by pernicious habits.

The subject of faulty breathing, however, is not a new one, and its ill effects are clearly recognised by those who have given the matter much attention. Those who do not understand its importance, we, as medical men, should instruct, and as Sidney Smith has well said in regard to any new discovery, "say it so long and so loud as to compel mankind to hear us."

Mouth-breathing in every instance is followed sooner or later by more or less ill effects, but these ill effects are much sooner produced when the person participates in active exercise, which greatly accelerates the action of the heart and lungs, than when in a quiescent condition; and in no athletic exercise are great demands more frequently made on these organs, and the ill effects of mouth-breathing more pronounced, than in bicycling.

This observation is made from the large number of persons who as patients have complained to the writer of the distress which they experienced from dryness and irritability of the throat while riding their wheel, caused by mouth-breathing, necessitated by obstructed nostrils. Two of this number were physicians, and one a D. D. S., who were obliged to limit their use of the wheel on this account.

The normal capacity of the nasal passages for the transmission of air is equal to that of the larynx when widely opened. There-

1. Read before the Medical Association of Central New York, October 20, 1896.

fore, provided there are no obstructions in the naso-pharynx and fauces, such as adenoid growths or enlarged tonsils, there should be no necessity for opening the mouth to breathe during active exercise.

The capacity of somewhat contracted nasal passages may be, during the ordinary habits of life, adequate for the transmission of sufficient air for the perfect aeration of the blood ; but during active physical exercise the worn-out material of the tissue is so greatly increased, that an unusually large amount of oxygen is required in order fully to oxygenize this effete material which is taken up by the blood. Therefore, if sufficient air cannot pass through the nasal passages for the proper oxygenation of the blood, it necessarily must find ingress through the only other avenue left—the mouth.

In such cases, then, the greater the amount of air breathed the greater is the amount which of necessity passes through the mouth. Thus in a short time the habit of mouth-breathing is acquired, and sooner or later the person will be found nearly all the time, particularly when asleep, breathing through the mouth. The nose, like a disused roadway which soon grows up with weeds and bushes, becomes still further occluded by thickened and engorged tissues, from lack of proper use.

Man was intended to be a nose-breather only. This is clearly shown by the functions which the nose performs during respiration. It is also shown by the fact that the first inspiration of the new-born babe is through the nostrils; and if these passages are closed, the breathing is very greatly embarrassed, even though the mouth be opened. Cases are known of new-born infants suffocating because the nostrils were occluded. That the air enters the nostrils before it reaches the lungs was shown by Cassels.[2] He demonstrated this fact by cases in which he found air in the tympanic cavities of still-born children when no air could be found in the lung tissue.

Not only is man a nose-breather, but also the lower animals—many of them by necessity, as they are not provided with communication between the mouth and the respiratory passages. This we find to be the case with the solipeds.

There are several important functions which the nose performs in fitting the air for respiration. These are : (1) it serves the purpose of a filter for arresting foreign substances, such as dust and germs ; (2) it imparts moisture to the air ; and (3) it modifies the temperature of the air, adapting it for respiration.

(1). That the nose performs the functions of a filter is very clearly shown by the large amount of dust and other foreign substances found in the secretions when the person has been inhaling air laden with these substances. It is also shown by the large number of germs deposited in the nose and not found in the air-passages below, when the air is respired through the nasal passages. St. Clair Thompson and Hewlett,[3] of London, have shown by their researches that at least 1,500 microörganisms are inspired every hour, while the number in the average London atmosphere sometimes rises to 14,000. As has been shown by Tyndall,[4] Gunning,[5] Strauss and Dubreuil,[6] the expired air is, nevertheless, practically free from them. In the experiments of Strauss, of 600 microbes contained in the air inhaled, a single one only was expired. Litster[7] has observed, in connection with the treatment of pneumothorax, that microörganisms inhaled do not reach the air cells, or the pleural cavity, if inflated with air which has found its way through a wounded lung, while Hildebrandt[8] shows by his experiments that they are arrested in the nasal passages. This is also confirmed by St. Clair Thompson and Hewlett,[9] by the examination of the tracheal mucus from recently killed animals, which is always found sterile. Furthermore, these organisms are mainly arrested in the anterior portion of the nose, since the vibrissæ of the vestibule are found swarming with them, while the deeper portions of the nose are comparatively free from them.

The scarcity of microörganisms in the deeper portions of the nose is also shown by St. Clair Thompson and Hewlett to be due, first, to the active expulsive powers of the ciliated epithelium of the mucous membrane, which has the power to move particles along on its surface at the rate of 25 millimeters per minute; and, secondly, to the special properties of the nasal mucus, which renders it an unsuitable soil for the growth of microörganisms, and hence does not favor their multiplication.

(2.) It imparts moisture to the air. It is estimated by physiologists that during twenty-four hours about 5,000 grains of water are taken up by the respiratory current of air in its passage through the respiratory apparatus. Now, Bosworth[10] maintains that the greater portion of this water is derived from the nasal passages, for the reason that the vascular tissue covering the turbinated bodies is the only tissue capable of furnishing it, and that the mucous membranes of the lower air-passages contain mucous glands only, which secrete mucus alone. Numerous experiments have been made which confirm these observations. Aschenbrodt[11] found that each five liters of air passed from an air respirator into one nostril and out of the other, took up 18 grammes of water. By computing the result for twenty-four hours we have 5,000 grains of water, the amount found by other experimenters in the air expired during twenty-four hours. This large amount of water is therefore imparted to the air during its passage through the nose.

The amount of water given off by this vascular tissue is furthermore controlled by the vasomotor nerves, which regulate the supply according to the varying degrees of humidity of the air respired.

(3). The modification of temperature that takes place in the nose is especially important when the person is subjected to extremes of heat and cold. Air on passing through the nose is brought very nearly to the bodily temperature, regardless of the temperature of the air before inhalation.

The heating power of the nasal passages was also ascertained by Aschenbrodt with an apparatus specially constructed for the purpose, in a manner similar to that in which he showed the amount of watery vapor imparted to the air as it passes through the nose. Air made to pass in through one nostril and out the other, the passage to the pharynx being closed, was found, in the different experiments, to be raised to a comparatively uniform temperature of from 86° to 92.3° F. All these experiments regarding the moisture and heat have been confirmed by Kayser[12], Bloch[13], and Greville Macdonald[14]. The latter also showed that air at an exceedingly high temperature, as at 140°, is reduced to the uniform temperature of 92° on passing through the nose alone.

With a knowledge of the important functions which the nose performs in purifying and modifying the air in fitting it for respiration, the injurious effects of mouth-breathing upon the throat and lungs can very readily be understood. Air passing directly to the lungs through the mouth has no filter by which the dust and germs are removed, nor is the air modified by having moisture and warmth imparted to it. In consequence of this, dryness of the pharynx is soon produced and the intractable disease termed pharyngitis sicca is engendered. The larynx becomes irritated, hoarseness and coughs are induced thereby, and a general catarrh of the throat and bronchi is caused. These conditions, extending into the deeper air-passages, soon produce an excellent feeding-ground for bacilli to develop, and consumption is not the infrequent result.

Moreover, mouth-breathing gives a peculiarly vacant expression to the countenance and the open mouth of the mouth-breather is so conspicuous that already it has come to be recognised by the laity and commented upon by the press as the "bicycle mouth," owing to the frequency with which it is seen among bicyclists.

These evil effects of mouth-breathing are not, however, confined to cyclists[15]; but it is equally injurious to all who from carelessness or from necessity, by reason of the nostrils being narrowed or obstructed by diseased conditions, it becomes a habit or practice. When this condition of mouth-breathing exists, however, the inhalation of a large amount of air, which is necessitated when active exercise is participated in, as in wheeling, accentuates the ill effect of this perverted respiration, and to a great degree lessens the benefits derived from this splendid exercise. Likewise, and as the natural means of affording relief from these more or less dangerous consequences, there is emphasised the importance of giving proper attention to the nasal passages and rendering them free and unobstructed.

Respirators worn over the mouth, so often recommended a few years ago to patients suffering from throat and chest affections, have been abandoned since the functions of nature's respirator, the nose, have been more fully understood.

It is also a well-known fact that the endurance of athletes or persons engaged in any active exercise, other things being equal, is proportionate to the freedom of respiration through the nose and the proper oxygenation of the blood.

The reason that we direct our warning especially to cyclists is that cycling is so nearly universal at the present time as to render it the principal mode by which exercise is taken, and therefore the ill effects of mouth-breathing are most often noticed among wheelmen. It is estimated that four hundred thousand in Great Britain and over a million in this country participate in cycling. The larger the number, the more urgent becomes our duty to warn them of the evil consequences of mouth-breathing, and to teach them the importance of obeying Catlin's injunction, Shut your mouth and save your life !

REFERENCES.

2. Edinburgh Medical Journal, February, 1877, p. 730.

3. "The Fate of Microörganisms in Inspired Air." Lancet, London, 1896. Vol. I., p. 86.

4. Pathological Transactions of the Royal Society, 1876. Vol. CLXVI., p. 27.

5. Klinische Monatsblätter für Augenheilkunde, 1882.
6. La Semaine Medicale, 1887. No. 49, p. 493.
7. British Medical Journal, July 18, 1868.
8. Beitrage zur Pathologisches Anatomie und Physiologie, Ziegler und Nauwerick, 1888. Bd. II., p. 421.
9. Op. Cit., p. 86.
10. New York Medical Journal, 1886. Vol. XLIII., p. 464.
11. Ueber die Bedeutung der Nase im Respiration, Wurzburg, 1886.
12. Pfleuger's Archives, 1887. Vol. XII., p. 127-147.
13. Archives of Otology, 1888. Vol. XVI., p. 279.
14. Diseases of the Nose, London, 1892, p. 4.
15. Public speakers, singers and persons who use their voice much, and who, while using it, carelessly inspire through the mouth, instead of through the nose, suffer in like manner from irritation of the throat and larynx from this cause. The disease termed clergyman's sore throat more often results from this cause rather than from the use of the throat, as commonly supposed. All persons during conversation, laughing and the like, take in more or less air through the mouth without detriment, but excessive and prolonged breathing through the mouth will, sooner or later, in every instance, produce its ill effects on the throat and the respiratory organs below. The general health suffers also in a corresponding degree from the insufficient amount of air inhaled by mouth-breathers.
16. "Cycling in Health and Disease." British Medical Journal, 1896. Vol. I., p. 1158.

28 North Clinton Street.

